# Antibody-drug conjugates: the paradigm shifts in the targeted cancer therapy

**DOI:** 10.3389/fimmu.2023.1203073

**Published:** 2023-08-21

**Authors:** Devesh Aggarwal, Jie Yang, Md. Abdus Salam, Sagnik Sengupta, Md. Yusuf Al-Amin, Saad Mustafa, Mohammad Aasif Khan, Xun Huang, Jogendra Singh Pawar

**Affiliations:** ^1^ Department of Chemistry, Purdue University, West Lafayette, IN, United States; ^2^ Department of Orthopedic Surgery, Qilu Hospital, Cheeloo College of Medicine, Shandong University, Jinan, Shandong, China; ^3^ Department of Basic Medical Sciences, Kulliyyah of Medicine, International Islamic University Malaysia, Kuantan, Malaysia; ^4^ Purdue University Interdisciplinary Life Sciences Graduate Program, Purdue University, West Lafayette, IN, United States; ^5^ Deen Dayal Upadhyaya (DDU) Kaushal Kendra, Jamia Millia Islamia University, New Delhi, India; ^6^ Division of Hematology and Medical Oncology, Department of Medicine, University of Texas Health Science Center at San Antonio (UTHSCSA), San Antonio, TX, United States; ^7^ Shandong Provincial Key Laboratory of Detection Technology for Tumor Markers, College of Medicine, Linyi University, Linyi, Shandong, China; ^8^ Department of Medicinal Chemistry and Molecular Pharmacology, Purdue University, West Lafayette, IN, United States; ^9^ The Ohio State University Comprehensive Cancer Center – Arthur G. James Cancer Hospital and Richard J. Solove Research Institute, Columbus, OH, United States

**Keywords:** Antibody-drug conjugate (ADC), targeted therapy, cancer chemotherapy, payloads, warheads

## Abstract

Cancer is one of the deadliest diseases, causing million of deaths each year globally. Conventional anti-cancer therapies are non-targeted and have systemic toxicities limiting their versatile applications in many cancers. So, there is an unmet need for more specific therapeutic options that will be effective as well as free from toxicities. Antibody-drug conjugates (ADCs) are suitable alternatives with the right potential and improved therapeutic index for cancer therapy. The ADCs are highly precise new class of biopharmaceutical products that covalently linked a monoclonal antibody (mAb) (binds explicitly to a tumor-associated surface antigen) with a customized cytotoxic drug (kills cancer cells) and tied via a chemical linker (releases the drug). Due to its precise design, it brings about the target cell killing sparing the normal counterpart and free from the toxicities of conventional chemotherapy. It has never been so easy to develop potential ADCs for successful therapeutic usage. With relentless efforts, it took almost a century for scientists to advance the formula and design ADCs for its current clinical applications. Until now, several ADCs have passed successfully through preclinical and clinical trials and because of proven efficacy, a few are approved by the FDA to treat various cancer types. Even though ADCs posed some shortcomings like adverse effects and resistance at various stages of development, with continuous efforts most of these limitations are addressed and overcome to improve their efficacy. In this review, the basics of ADCs, physical and chemical properties, the evolution of design, limitations, and future potentials are discussed.

## Graphical Abstract

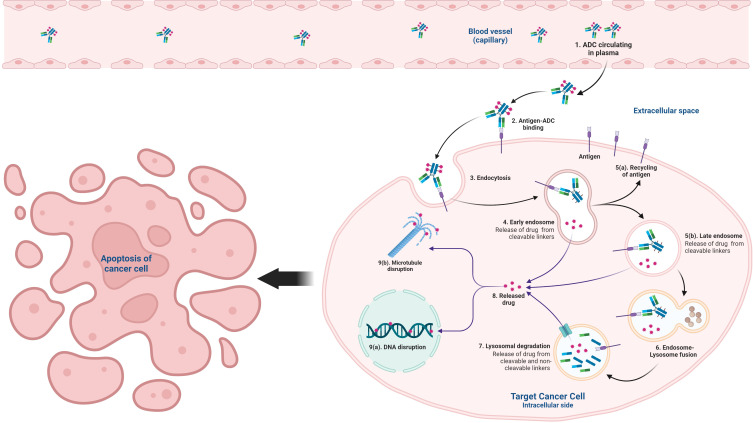

## 1 Introduction

Cancer remains the second leading cause of death reckoning million of deaths globally each year. An estimated 1,958,310 new cancer cases and 609,820 cancer deaths are projected in 2023 only (1, 2). Treatments for cancers include surgery, chemotherapy, radiotherapy, immunotherapy, stem cell therapy, laser treatment, hyperthermia, and photodynamic therapy among others (3–5). Chemotherapy is the most common therapeutic intervention for cancer among these options (4–6). However, chemotherapy is associated with toxic side effects, resistance and may not work well for many patients. The unsatisfactory results, off-target toxicities, and poor prognosis are major limiting factors for chemotherapy in its clinical applications. To improve the efficacy of chemotherapy, relentless efforts have been made by using potent cytotoxic agents either single or in combination (3, 5, 7). Enhanced understanding of cancer biology in recent years has allowed a paradigm shift in cancer treatment from traditional chemotherapy to targeted therapies by exploiting the biology of tumor cells. There have been several attempts to overcome the issues related to conventional chemotherapy. Antibody–drug conjugate (ADC) was conceived as a novel concept to bridge the gap between the monoclonal antibody (mAb) and cytotoxic drugs for the improved therapeutic window. ADCs target neoantigens that are self-antigens generated by the mutations in tumor cells and are exclusively expressed by the tumor cells. However, there are alternative ways by which neoantigens can be produced like viral infection, alternative splicing and gene rearrangement. These neoantigens are ideal targets for T cells’ recognition of cancer cells and to stimulate strong anti-tumor immune response. Vaccines developed against neoantigens are now being used in clinical trials in various solid tumors (8).

Neoantigens can be classified into two categories: shared neoantigens and personalized neoantigens. Shared neoantigens refer to mutated antigens that are common across different cancer patients and not present in the normal genome. Personalized neoantigens on the other hand refer to mutated antigens that are unique to most neoantigens and completely different from patient to patient. Thus, the personalized neoantigen drug preparation can only specifically target an individual patient, i.e., personalized therapy (9). There are two approaches exist which use antibodies targeting neoantigens. Firstly, antibodies can bind to cell surface major histocompatibility complex calss I (MHC-I)-presented peptides, derived from intracellular proteins, called T-cell receptor mimics (TCRm) antibodies or TCR-like antibodies. Secondly, intracellular antibodies also called intrabodies that can be produced inside the tumor cells that inhibit the functions of oncogenic proteins (10). Major histocompatibility complex (MHC)-bound peptides that arise from tumor-specific neoantigen mutations, are promising targets for adoptive T-cell therapy with autologous tumor-infiltrating lymphocytes expressing endogenous TCRs, gene-modified T cells expressing novel T-cell receptors or chimeric antigen receptors (CAR) T cells comprising recombinant antibodies against extracellular cell surface molecules or TCR-like antibodies. On contrary, intracellular neoantigens are important targets for intrabodies (11). Innovation of ADCs is the revolutionary approach for improved and targeted drug delivery to cancer patients and a milestone addition to the arsenal of cancer chemotherapies (12).

ADCs are an amalgam comprises of a monoclonal antibody (mAb) conjugated to the potent cytotoxic payload via a chemical linker (13–15). It is a unique biopharmaceutical approach to achieving targeted delivery of cytotoxic drugs only to the cancer cells guided by the mAb against the tumor-associated antigen expressed on the cancer cell surface. Due to its high precision, the improved therapeutic window can be achieved only for the cells that express the target antigen sparing the normal cells, thus can minimize the systemic toxicities. ADCs are taken via receptor-mediated endocytosis by the tumor cell after specific antigen-antibody interaction and processed subsequently in the cytosol through the endo-lysosomal pathway. Breakage of the chemical linker between the payload and mAb allows the release of the drugs into the cytosol to cause eventual cell death via its cytotoxic action (15, 16). There are two types of chemical linkers; cleavable (that utilizes the inherent properties of the tumor cells for selective release of cytotoxins from ADCs) and non-cleavable (relies completely on the lysosomal proteolytic degradation of mAb to release cytotoxins after internalization), however, the overall mechanisms are still similar. After internalization, drugs can cause cytotoxicity by various mechanisms of action, including DNA damage or inhibition of microtubule assembly depending on the payload. Although the concept seems simple, the combination of three components of ADCs (mAb, linker, and cytotoxin) into an optimized and functional therapeutic agent remains a great challenge. The usefulness of an ADC is based on the function of intricate interactions of payload, linker, and monoclonal antibody-specific variables of the ADC and numerous tumor integrant and the tumor microenvironment (TME) (16–18). Other mechanisms like the “Bystander effect” also contribute to its cytotoxic activity. Bystander effect refers to the phenomenon when the design of ADCs permits diffusion of the payload drug from an antigen bearing cell to the adjacent cells that lack the target antigen and thereby kill those heterogeneous tumor cells. It is one of the important criteria to be considered while using ADCs in the management of heterogeneous tumors (18, 19). Even though initial preparations of ADCs posed some limitations at various stages of development, with continuous efforts most of these limitations are addressed and overcome to improve their efficacy and currently a few ADCs are approved by the FDA for clinical use after successful pre-clinical and clinical trials (12, 17, 20). In this comprehensive review, a brief discussion on the basic principles of ADCs, their success, limitations, and future promises are highlighted.

## 2 Timeline of ADCs development

The concept of ADCs has been mostly attributed to the idea of a ‘magic bullet’ conceived by German physician and scientist Paul Ehrlich in the 1900s, who suggested targeted delivery of toxic agents against microbes and tumor cells using antibodies (21, 22). It took almost four decades of research to visualize some promising outcomes of ADCs when through improvement in chemistry a cytotoxic drug called methotrexate was linked successfully with polyclonal rodent immunoglobulins directed towards leukemic cells in the 1950s (23). It took a while to get the first antibody-conjugated drug and it wasn’t until the 1980s when the first ADC was introduced in the clinical trials using mouse IgG (24–26). This was soon followed by the development of chimeric and human mAbs in the 1990s (27–29). In May 2000, FDA approved the first ADC, gemtuzumab ozogamicin (Mylotarg), a humanized monoclonal antibody (gemtuzumab) conjugated with drug (ozogamicin) targeting CD33 protein, made by Wyeth for the treatment of patients with acute myeloid leukemia (AML) (30, 31). However, gemtuzumab ozogamicin failed to enhance overall survival and was associated with a greater risk of lethal toxic side effects during the post-approval trial when compared to chemotherapy alone, prompting Wyeth (acquired by Pfizer) to pull back this ADC voluntarily in 2010 from the market (32).

The second generation of ADCs targeting other key cell surface antigens with new more potent warheads are also being discovered. In 2011, brentuximab vedotin (developed by Seattle Genetics) consists of a chimeric monoclonal antibody linked with a peptide-based (valine-citrulline) cleavable linker targeted to CD30 (also known as TNFRSF8) cell surface protein was launched for the treatment of relapsed or refractory Hodgkin lymphoma and in 2013, ado-trastuzumab emtansine (also known as T-DM1, developed by Roche) targeting human epidermal growth factor receptor 2 (HER2; also known as ERBB2, Kadcyla), was commercialized for the treatment of solid tumors (33–36; 37). IgG1 isotype mAb has been used for both of these two ADCs, which is superior to the IgG4 isotype for bioconjugation with small-molecule payloads and high targeting ability.

The third-generation ADCs include polatuzumab vedotin (Polivy, Roche), enfortumab vedotin (Astellas Pharma and Seagen), and fam-trastuzumab deruxtecan (Enhertu, Daiichi Sankyo) that utilized fully humanized mAb instead of chimeric mAb to reduce immunogenicity and better pharmacokinetics.

A significant number of ADCs have been developed and capitalized since their first launch in 2000 and until now there are 12 FDA-approved ADCs available for both hematological malignancies and solid tumors worldwide. Still, more than 100 ADC candidates are now under investigations in different phases of clinical trials (12, 17, 20). Although the rates of ADC development appear to be picking up, with more ADCs being approved in 2019 and 2020, still ADC is an emerging class of novel biopharmaceuticals that combine the principles of both chemotherapy and immunotherapy.

## 3 Structure of ADC

**Figure 1:**
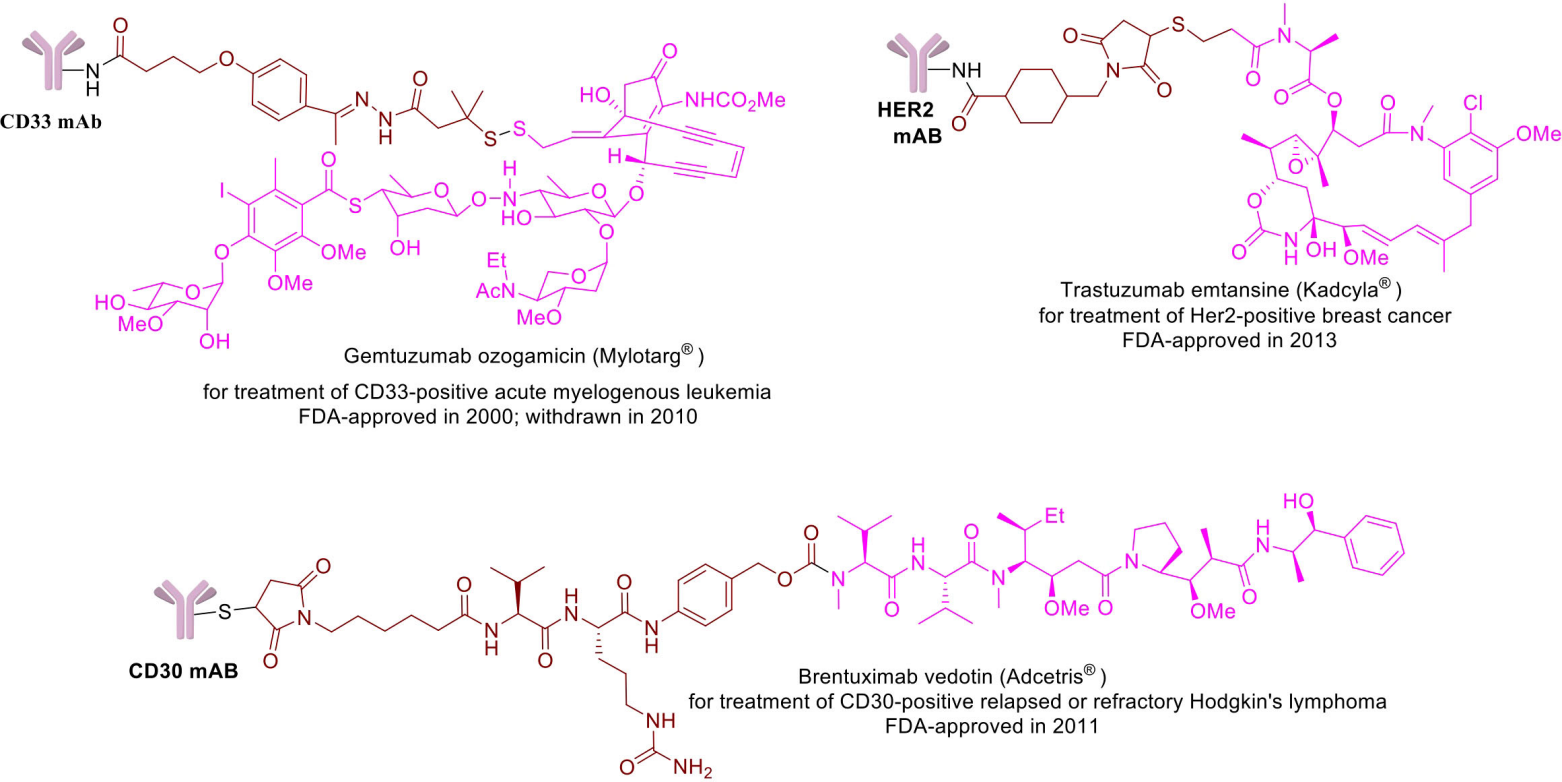
Chemical structures of a few example FDA-approved ADCs: Mylotrag^®^, Adcetris^®^, Kadcyla^®^ (pink: payload, brown: linker).

ADC is a hybrid molecule that combines biologics as components. It is made up of an antibody scaffold that has been modified and is covalently connected by a chemical linker with small molecular payloads that vary in number. ADCs comprise of three core components: a monoclonal antibody specific to a tumor-associated antigen, a connecting linker, and the cytotoxic payload (**Figure 1**). Each core component can vary greatly amongst ADCs, having a significant impact on their pharmacological and clinical characteristics. Moreover, each component has complex interactions with the tumor and the tumor microenvironment (TME).

### 3.1 Antibody-Antigen selection

The discovery of the specific antigenic determinant for the generation of mAb component against it is one of the most fundamental tasks in designing an ADC for cancer therapy. As for targets in antibody-based treatment, there are 328 distinct antigens including ERBB2, CD19, CD33, CD22, and MSLN (human mesothelin) are the frequently targeted antigens along with approximately 50 other well-defined target antigens (38, 39). There are several conditions that must be considered before choosing a target antigen. First, there should be abundant expression of the target antigen only in the tumor cells while the healthy cells will be negative for this antigen or express minimal. Second, it should be a cell surface antigen so that circulating mAb can recognize and binds with it easily. Third, to facilitate the internalization of ADC and to enhance the efficacy of cytotoxic drugs, the target antigen should possess the property of receptor-mediated endocytosis. For example, expressions of HER2, TROP2, EGFR, EpCAM, and nectin 4 are also observed to some extent in non-malignant cells, however because of their overexpression in tumor cells has made them suitable as target antigens in designing ADCs for the solid tumors and currently approved by the FDA (40–44).

Antibodies have many exciting naturally favorable attributes such as specificity, potency, and metabolic stability, which have made them versatile molecules for the antibody-based therapies including ADCs. All naturally occurring antibody fragments and engineered bispecific mAb provide intriguing prospects for their use in antibody-based therapies, but immunoglobulin G (IgG) dominates the current pool of biologics including ADCs (45). First generation ADCs utilized heterologous mAb from murine, which elicited a significant immunological reactions with formation of anti-human antibodies in many recipients resulting decreased therapeutic effectiveness (46). Due to early difficulties with the use of murine antibodies, chimeric or humanized mAb were incorporated as backbone in second generation ADCs, which substantially reduces the burden of immunological reactions but does not fully eliminate. There are four subclasses of human IgG (IgG1, IgG2, IgG3, and IgG4) based on their constant region domains and hinge regions, and subtle structural differences among these subclasses influence in their biological functions of mAb for solubility, half-life, as well as their binding capacity for various Fc receptors (FcRs) present on immune effector cells (47). Due to its superiority in terms of structural homogeneity, long half-life, potent effector function, and thorough characterization, IgG1 mAb dominates as the backbone of majority of ADCs preparations (e.g., brentuximab vedotin, ado-trastuzumab emtansine). Therapeutics based on mAb that require antigen binding without extensive immune activation depend on IgG2 and IgG4 subclasses and IgG4 has been utilized as backbone for gemtuzumab ozogamicin and inotuzumab ozogamicin (48–50). Antibodies also induce immune response via Fc domain while interacting with Fcγ receptors (FcγRs) on immune cells, inducing cytocynines release, cell death by complement-dependent cytotoxicity (CDC) and antibody-dependent cell-mediated cytotoxicity (ADCC) and thus increase cytotoxicity and side effects. Glyco-engineered antibodies with abolished FcgR interaction are now more and more used. This glycosylation completely switches the antibody effector interaction (Fc domain interaction with FcγRs) and imparts a novel approach for developing therapeutic antibodies without any ADCC and CDC activities with certainly low immunogenicity (51).

Another concern with antibodies is to understand their extracellular modes of action in killing the tumor cells like CDC, ADCC or antibody-dependent cell-mediated phagocytosis (ADCP) (12, 17, 20).

Thus, while designing and constructing an ADC molecule, the relative roles of the cytotoxic agent and the antibody in the anti-tumor activity and toxicity profiles of the entire ADC are taken into consideration. To optimize the antibody and antigen selections, biopharmaceutical researchers are striving continuously with various approaches for improving efficacy of the newer generations of ADCs.

### 3.2 Warheads/payloads

ADCs are engineered therapeutics that require elaborate technical capabilities and highly potent chemical substances, specifically, the multi-step process of production of cytotoxic payloads. After being internalized into the cytosol of a tumor cell, ADC releases the cytotoxic payload or warhead for its action to be mediated on the target cell. The efficacy of warheads ideally should be sufficient to destroy tumor cells even at low dosages (IC_50_ range of approximately 10^−10^-10^−12^ M) due to limited delivery (20, 52). ADCs now in clinical studies employ just a few numbers of cytotoxic chemical families as warheads. Initial ADCs were designed to deliver known anticancer chemotherapeutic drugs such as methotrexate, doxorubicin, alkaloids and more. Unfortunately, those ADCs were merely more effective than traditional equivalents and sometimes required extremely high dosage for their action which increased the risk of systemic toxicities (12). Moreover, the amount of payload available to the tumor cells was not sufficient to cause significant cytotoxicity with the main challenge being the TME (12, 17). These findings prompted further research into ADCs including very powerful chemotherapeutic agents such as auristatins and maytansinoids (tubulin inhibitors), and calicheamicin analogues (DNA-damaging agents), which have lethal effect at sub-nanomolar doses (53–56). There is another class, called camptothecin (CPT), a topoisomerase inhibitor with its notable analogues, the exatecan derivative (DXd) and the irinotecan metabolite (SN-38) that results in DNA breaks (20, 57). Although all these drugs are being used widely in cancer chemotherapy, none of these payloads have been proven to be acceptable for systemic administration as free drugs. It had been a few decades that majority of these drugs were discovered, but their research was halted due to a restricted therapeutic index. Conjugation into ADC allows delivery of these drugs directly to the tumor cells concealing their cytotoxic effects in the circulation thus greatly lowering the systemic toxicities.

Physical properties of these drugs are also critical for ADCs functioning (12, 18, 20). Due to hydrophobic nature, most of these cytotoxic drugs employed in ADCs are likely to cause antibody aggregation, that must be prevented not only to maintain an extended shelf life but to minimize rapid clearance rates and immunogenicity. Further, the drugs should keep its efficacy once changed for linking and should have appropriate water solubility as a conjugate to be stable in aqueous formulation. Furthermore, the drugs must be available for synthetic preparation and attainable through a cost-effective technique under acceptable manufacturing practice standards. In the context of ‘bystander effect’, the hydrophobicity of the detachable payloads is also considered to be an essential component to minimize the cytotoxicity of adjoining healthy cells due to diffusion of cell permeable payloads from the target cells (19, 58).

### 3.3 Linker

The biological molecules known as linkers used in ADCs are for the linkage between the antibody and the warhead. Effective linkers must ensure ADC stability in the circulation as well as efficient cleavage upon internalization by the tumor cells. Since premature drug release into the blood might increase in systemic toxicity with consequent reduced therapeutic index, effective linker design must achieve an equilibrium between the necessity for long-term stability in the circulation and effective cleavage upon internalization into the target cell (59, 60). There has been significant progress in linker technology since the beginning of ADC research, which has contributed enormously to the clinical achievements of ADC formulations until now (60). For linkers, there are both non-cleavable and cleavable linkers that ensure the requirements of ADC design mentioned above. Non-cleavable linkers are made up of stable bonds e.g., non-reducible thioether linkage (SMCC), maleimidocaproyl linkage etc. that can withstand the proteolytic degradation inside the cytosol and release of cytotoxic payloads only occurs after antigen-specific internalization of the conjugate and full breakdown of mAb by lysosomal proteolytic degradation (12, 14, 20). Thus, the resultant release of the cytotoxic drugs is linked to the antibody’s linker and an amino acid residue, which specifically kills tumor cells and doesn’t release at non-target locations to cause any damage to healthy cells. Additionally, the non-cleavable linkers have provision to modify the chemical characteristics of small molecules to enhance the affinity of the transporter and increase its effectiveness. Although they have lesser membrane permeability, these linkers are more stable than cleavable ones and increase the therapeutic window by minimizing the off-target toxicities. Examples of non-cleavable linkers include thioether linkers (used in T-DM1) and maleimide-based linkers (used in belantamab mafodotin) (61).

**Figure 2:**
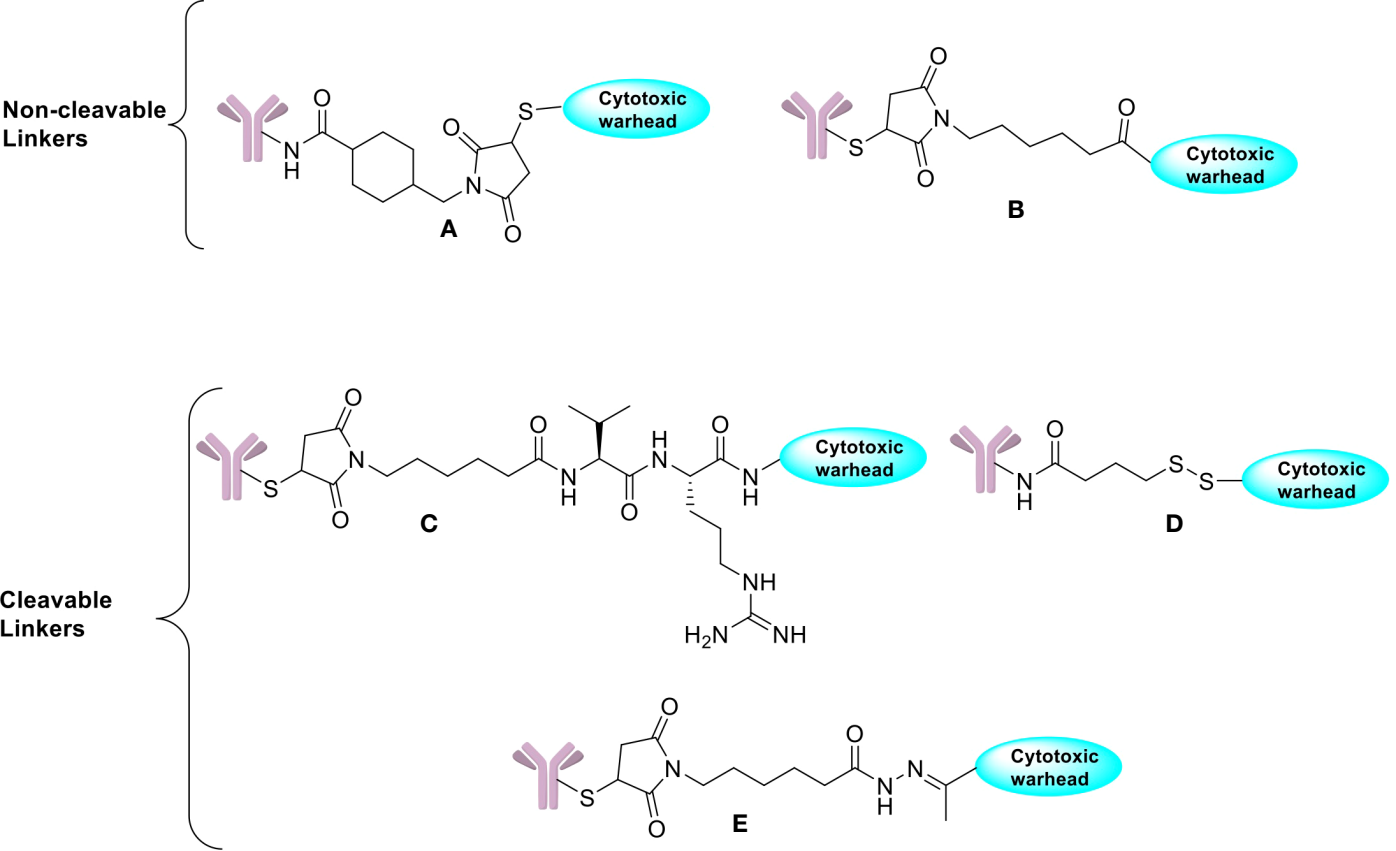
Chemical structures of non-cleavable and cleavable linkers. **(A)** SMCC linker. **(B)** Maleimidocaproyl linker. **(C)** Protease cleavable peptide-Based Linker. **(D)** Reducible disulfide linker. **(E)** Acid-sensitive hydrazone linker.

Cleavable linkers on the other hand depend on the inherent properties of the tumor cells in selective release of cytotoxic payloads from ADCs and can be influenced by external pH (acid-labile linkers), particular lysosomal proteases (protease-cleavable linkers), or glutathione reduction (disulphide linkers). Use of cleavable linkers is a more traditional approach where the cytotoxic drug is intended to be released after being processed inside the cell. Examples of cleavable linkers-based ADCs include: gemtuzumab ozogamicin (pH-sensitive hydrazone linkers), sacituzumab govitecan, brentuximab vedotin, polatuzumavedotin, enfortumab vedotin, and trastuzumab deruxtecan (protease-sensitive linkers) and mirvetuximab soravtansine and indatuximab ravtansine (glutathione-sensitive linkers) (20, 60). Even though they are specifically designed to maintain stability during circulation, it has been suggested that nonspecific drug release may occur in case of acid cleavable linkers, which can be overcome by using non-cleavable linkers. Both types of linkers are featured in the FDA approved list of ADCs with their own advantages (**Figure 2**).

### 3.4 The hybrid

After selecting all the three major components of an ADC, the overall structure also plays a role in its function. It is important to note that, selection of all three right components is tricky and any discrepancy can lead to different biophysical and functional properties of the resultant ADC (13, 16, 20, 62, 63). For example, variations in the linker could have a tremendous impact on the ADC both for biophysical and functional properties. There are two primary conjugation methods for connecting linkers to antibodies, leading in either varied or site-specific payload placement. Currently, heterogeneous conjugates dominate in ADCs that are in the clinical pipeline, despite efficient understanding of analytical benefits of site-specific conjugation. Heterogeneous ADCs have dominated the field for a long time because the methods for creating them are relatively straightforward. However, in recent years, there has been a shift towards the development of site-specific ADCs, which have a uniform drug-to-antibody ratio (DAR) and specific conjugation sites. These site-specific ADCs are produced using engineered antibodies with specific conjugation sites or using enzymatic conjugation methods. Heterogeneous conjugation is dominant to site-specific conjugation for several reasons, at least: older, well known, more rapid, less expensive, not limited by a patent protecting the site-specific method. It is an active area of research to determine which approach will ultimately prove most effective in different therapeutic contexts (64).

In constructing an optimum hybrid, the DAR of ADCs is a critical attribute for their therapeutic efficacy and pharmacokinetics. Stability, solubility, antigen binding, clearance and biodistribution of an ADC can be influenced by the number of DAR in its configuration. In order to achieve adequate cytotoxicity by ADC, a certain number of linker–drug units need to be attached to each mAb.

DAR is a variable trait that represents the average number of payload moieties attached to each mAb. The value of DAR in ADCs has important attributes for both pharmacokinetics and pharmacodynamics of cytotoxic drug used in it. The range of DAR varies between 2 to 8 for optimum antitumor activity *in vivo* and this range is currently approved for ADCs preparations (12, 20). High DARs have been positively correlated with *in vitro* potency, however there was increased plasma clearance as demonstrated in preclinical models. To limit tumor ADC exposure and gaining similar activity to ADCs, lower DARs ranging from 1-2 have been used (e.g., brentuximab vedotin) but due to biotransformation of ADCs other heterogeneity can develop (65).

High-DAR ADCs may have poor biophysical properties that limit effectiveness and increase toxicity (66, 67); however, these shortcomings can be reduced by employing certain conjugation and linker maneuvers. Pre-clinical data infer that rapid hepatic clearance due to high DARs was linked to increased hydrophobicity of the antibody–linker complex, which may be avoided by employing hydrophilic constructions (e.g., higher DARs used in sacituzumab govitecan are directly associated with greater anti-tumor activity *in vivo* without influencing plasma clearance) (62, 68). The hydrophobicity of the detachable payloads is also believed to be a key component, especially in terms of the “bystander effect.”

## 4 Toxicity

Innovations of ADCs were primarily aimed to reduce the toxicities associated with conventional chemotherapeutic agents by better tumor targeting; nevertheless, significant adverse events (AEs) were seen in several early studies using ADCs (12, 69, 70). The majority of clinical experience on AEs has been with ADCs containing antimitotic payloads, which cause significant organ toxicity in the liver. Much less is known about the therapeutic consequences of administering DNA alkylators, however clinical studies have indicated that they target the hematopoietic compartments. Unlike the traditional toxicity profiles for conventional chemotherapeutic drugs, ADCs have a different type of accumulation profiles like ‘on- target, off- tumor’ and ‘off- target, off- tumor effects which depends on various components of the ADCs.

Since mechanisms of action of ADCs are target antigen dependent, it can cause ‘on- target, off- tumor’ toxicity, which generally happens when the target antigen is also expressed by non- malignant tissue outside the tumor. For example, early 1990s discovery of ADC BR96-doxorubicin targets the Lewis Y antigen and was found to be highly active in mouse xenograft models of multiple tumor types, however, unlike in mice, this antigen is expressed in non-malignant human tissues, particularly the gastrointestinal tract and had similar toxicity profiles (29). Likewise, ADC containing bivatuzumab mertansine targeted against CD44v6, expressed by squamous cell cancers of head and neck was associated with skin damage in approximately 80% of patients (71, 72). Even with distinct payloads, pulmonary toxicity was observed in cases of HER2-targeted ADCs T-DXd and trastuzumab duocarmycin via an unexplained mechanism (probably by targeting HER2- expressing cardiomyocytes, yet to be shown) (34, 73, 74). Curiosities go further when different toxicity profiles were noted in clinical trial with ADC preparations like Brentuximab vedotin, Polatuzumab vedotin and Enfortumab vedotin that all have the same payload, linker structure and similar DARs (12, 63).

The second mode of toxicity, the ‘off- target, off- tumor’ still seems to be more prominent with ADCs despite the above-mentioned observations (63, 69). This is not surprising due to the facts that payload releases in the circulation can go to tissues other than tumor, or the TME can have impact along with the payload on relevant non-malignant tissues. This is usually correlated with release of the cytotoxic drug in the blood stream due to premature cleavage of the linkers. This issue can be overcome to some extent by the usage of non-cleavable linkers. Some drugs may even cause toxicity after being cleaved inside the cells by the ‘bystander effect’ due to diffusion from target cells and uptake by adjacent non-malignant cells (19, 58). Absorption of cytotoxic payload into non-malignant cells may also occur in case of target-independent ADCs by processes such as macro or micro-pinocytosis, or via binding to Fc receptors, compounding other toxicity difficulties (75, 76). Further, there are intriguing cases of different toxicity patterns generated by the same ADC depending on the tumor type under investigation (12). Spectra of different observations in toxicity profiles make the study of the toxicity profiles very difficult and demands further works to be done to reveal the true pictures. These difficulties highlight the importance of attentive monitoring, cautious selection of dosage and meticulous AE reporting while using ADCs in clinical trials.

## 5 Challenges in ADC therapy

With the approval of various ADCs for human use recently and many more in clinical trials, the field has shown a great promise in targeted cancer therapy, however there are significant major challenges that need to be overcome for the clinical success (20). The intricacy on the part of ADC functioning raises intriguing concerns about the connection between the molecular structure of ADC and its clinical activity at the macro-level along with toxicity profiles. The events of pathway in a classic model of ADC action involves steps such as, mAb attachment to the target specific antigen, internalization, linker breakage, and intracellular payload release. Although this model provides a useful broad outline, tangibility seems different that varies significantly across various ADC preparations. As discussed above, toxicity and construction of a purposive hybrid are among the major challenges with regards to clinical success of ADCs.

Unlike regular drugs, pharmacokinetic and/or pharmacodynamic modeling of ADCs are quite complex. The relative quantity of three integral components may differ amongst various ADCs, depending on linker strength and product quality that may alter drug delivery over time and adds more variability. Moreover, accurate measurements of pharmacokinetic and/or pharmacodynamic data for ADCs are challenging to obtain, especially in patient populations (77). However, it appears that a very little fraction of administered ADC dose ends up reaching tumor cells as evidenced from labelled mAb-based studies as well as mathematical models (52, 78–80). This in turn emphasizes the importance of payload potency in ADC design once again. Nevertheless, pharmacokinetic and/or pharmacodynamic modeling (PK/PD) of ADCs has been proven effective in some cases. Further improvements in current tools as well as new advancements in technologies for bioanalysis, PK/PD models can be more refined to improve ADC design for improved patient benefit.

Although due to its inherent properties, ADCs work better than their counterpart for cancer therapy, however, their durable responses are limited by development of resistance against the antibody component of ADCs by down-regulation/mutation of the target cell surface antigen or against payloads by up-regulation of drug efflux transporters.

Comprehensive data on the mechanisms of ADC resistance have yet to gather, preliminary data show that the complex mode of action of ADCs offers tumors a plethora of escape mechanisms at many key stages, involving internalization, recycling of target antigen by tumor cell, interaction of antibody, antigen, and ADC and release of payload. Some of the proposed mechanisms include: (i) inhibition of ADC docking on cancer cell due to downregulation of the target antigen by the cell, limiting payload release; (ii) recycling of endosome to the cell surface resulting in ADC ejection back to exterior before releasing payload (this may happen due to changes in lysosomal acidity, redox environment, or proteolytic activities); (iii) activation of drug efflux transporters like ATP-binding cassette (ABC) in tumor cells resulting in payload efflux, shielding cells from cytotoxic destruction (12, 16, 62). Additional research is required to understand and identify mechanisms of ADC resistance and to establish predictive biomarkers to enhance patient selection. Other challenges include antigen related drawbacks, like selection of the right antigen-antibody combo, differences of antigen expression profiles in murine model vs humans, and meticulous consideration in patient selection. Although ADCs are intended to discharge their payload within the tumor cells, most linker types can cause premature payload release. Furthermore, the implications of the bystander on non-malignant neighboring cells are yet to be known. Despite these obstacles, the findings support the notion that, usefulness of ADCs in treating cancers based on targeting the oncoprotein clearly has advantages over the conventional counterpart but certainly there is room for further refinement of ADC to achieve the best therapeutic results.

## 6 Success stories

Multiple ADCs from different pharmaceutical giants have already been approved by the FDA to date and there are many more in the pipeline, suggesting that the target, even after all these challenges, is achievable. The ADCs so far in use are targeted towards a specific cancer type and all have shown promising results. Regarding construction of hybrids, a diverse range of essential 3 components has been used for different ADCs depending upon their therapeutic requirement. A few of the ADCs are discussed here.

Gemtuzumab ozogamicin, a CD33-targeted drug, was approved by the US FDA in 2000 as the first ADC (31) for the stand-alone treatment of relapsed and/or refractory (R/R) acute myeloid leukemia, but it was withdrawn from the market by Pfizer voluntarily in 2010 because its effectiveness was seriously challenged by the adverse event (AE) profile. Unfortunately, a confirmatory Phase III trial did not show a clinical benefit of gemtuzumab ozogamicin in conjunction with chemotherapy than conventional chemotherapy alone and the rate of fatalities as a result of treatment-related toxicity was significantly higher (32). Then again in 2017, gemtuzumab ozogamicin was approved for human use with lower recommended dose, a different schedule in combination with chemotherapy or on its own, and a new patient population. The second generation of ADCs known as brentuximab vedotin (developed by Seattle Genetics) with target antigens being CD30 (also known as TNFRSF8) and human epidermal growth factor receptor 2 (HER2; also known as ERBB2) came into the market in 2011, followed by trastuzumab emtansine (also known as T-DM1 or ado-trastuzumab emtansine) in 2013, developed by Roche (20, 33, 36). New treatments have also been added to the repertoire over time for these two ADCs. Since 2013, the field has been quite active with 14 ADCs are currently in human use (12 FDA approved and one each in Japan and China) for different cancer therapies and more than 100 ADCs are currently in different stages of clinical development (20). As of April 2022, there are 1685 ADCs registered for clinical trials and the indications have expanded to include infections (e.g., HIV, lung disease), autoimmune diseases (spondyloarthritis, Alzheimer’s disease) and metabolic diseases (obesity, diabetes), etc.

The field has further progressed with the technological advancement in synthetic biochemistry including mAb manufacturing, linker chemistry, and addition of more new payloads. It is speculated that upcoming third generation ADCs will have better combination of antibodies, drugs, and linkers. The pace of ADC development appears to be picking up with one ADC approved is 2017, two in 2019 and three more in 2020 and several others in different stages of preclinical and clinical research (12). Sacituzumab govitecan is currently approved for treating refractory metastatic triple negative breast cancer (TNBC) irrespective of TROP2 expression; polatuzumab vedotin is approved for refractory or relapse (R/R) diffuse large B cell lymphoma, and belantamab mafodotin is approved for R/R multiple myeloma, both of which are categorical for tumors of B cell lineages. Given the fact that the clinical trials using ADCs for cancer treatment began only in the 1980s, the speed of development is enormous, and ADCs represent a rapidly increasing field in cancer therapy. Of course, there was hinge initially, various ADCs technologies developed over the past decade have widened the possibilities for designing new ADCs with new potentials.

## 7 Discussion

Chemotherapy remains as one of the principal treatment modalities to fight against cancer, however it comes with its own limitations. Ever since the idea of exposing patients to cytotoxic drugs was accepted, major efforts have been made to address the concerns of toxicity and mode of delivery of these drugs. The use of prodrugs or drug delivery vehicles are a pretty common practice nowadays, but it usually comes at an expense of efficacy and off target effects. ADCs provide a new niche where the intended drug is selectively and precisely delivered to the tumor cells by targeting a specific antigen which is either usually overexpressed in cancer cells or is not present in non-malignant counterpart, hence reducing the toxicity. The biological advancements and its fine tuning with chemistry over time is the main impetus behind the success of development and effectiveness of these drugs. Indeed, technological breakthrough and cutting-edge biopharmaceutical platforms have enabled ADC engineering with innovative linkers, advanced payloads, and humanized monoclonal antibodies towards development of a new generation of ADCs with high DAR and manageable bystander effects. In principle the unique concept of formulation of ADC shows a lot of promise and the success stories are enormous, still a number of limitations and challenges need to be addressed for optimization in therapeutic achievement.

Altogether there are 13 ADCs (**Table 1**) currently available for human use; approved by the FDA and several others in different stages of clinical trials. Most of the ADCs in current use target solid tumors with HER2, TROP2, and nectin-4 are examples of specific targets. TROP-2 and nectin-4 were initially added as the tumor targets for sacituzumab govitecan and enfortumab vedotin, meanwhile, it extended further to include HER2 as target for trastuzumab deruxtecan for better therapeutic index (20). Research has extended further to sort additional potential targets other than tumor-associated antigens, and proteins produced in the tumor microenvironment (e.g., CD25, CD205, and B7-H3) or by cancer stem cells (e.g., DLL3, ephrin-A4, PTK7, and 5T4) have shown promise for which particular ADCs are in clinical trials (81).

**Table 1:** List of various antibody-drug conjugates (ADCs) approved by the FDA.

ADC	Antigen Targeted	Antibody Isotype	Linker Type	Warhead	Mechanism of Action	DAR	Clinical Usage
Adcetris (brentuximab vedotin)	CD30	IgG1	Protease-cleavable	Monomethyl auristatin E (MMAE)	Binds to CD30 on Hodgkin lymphoma and anaplastic large cell lymphoma cells, internalizes, and releases MMAE to induce cell death	~4	FDA-approved for Hodgkin lymphoma and ALCL (Aug 2011)
Kadcyla (ado-trastuzumab emtansine)	HER2	IgG1	Thioether	Maytansine	Binds to HER2 on breast cancer cells, internalizes, and releases maytansine to induce cell death	~3.5	FDA-approved for HER2-positive breast cancer (May 2019)
Polivy (polatuzumab vedotin-piiq)	CD79b	IgG1	Protease-cleavable	MMAE	Binds to CD79b on B-cell lymphoma cells, internalizes, and releases MMAE to induce cell death	~2	FDA-approved for relapsed or refractory diffuse large B-cell lymphoma (April 2023)
Besponsa (inotuzumab ozogamicin)	CD22	IgG4	Acid-labile	Calicheamicin	Binds to CD22 on B-cell ALL cells, internalizes, and releases calicheamicin to induce cell death	~3.5	FDA-approved for relapsed or refractory B-cell ALL (Aug 2017)
Blincyto (blinatumomab)	CD19	BiTE (no Fc region)	N/A	N/A	Binds to CD19 on B-cell ALL cells and T cells, brings them into close proximity, and activates T cells to kill cancer cells	N/A	FDA-approved for B-cell ALL (Dec 2018)
Mylotarg (gemtuzumab ozogamicin)	CD33	IgG4	Acid-labile	Calicheamicin	Binds to CD33 on AML cells, internalizes, and releases calicheamicin to induce cell death	~2	FDA-approved for newly diagnosed CD33-positive AML in combination with chemotherapy and for relapsed or refractory CD33-positive AML (Sep 2017; 2000)
Trodelvy (sacituzumab govitecan-hziy)	Trop-2	IgG1	Acid-cleavable	SN-38	Binds to Trop-2 on cancer cells, internalizes, and releases SN-38, a topoisomerase inhibitor, to induce cell death	~7	FDA-approved for metastatic triple-negative breast cancer (2020)
Padcev (enfortumab vedotin-ejfv)	Nectin-4	IgG1	Protease-cleavable	MMAE	Binds to Nectin-4 on urothelial cancer cells, internalizes, and releases MMAE to induce cell death	~2.5	FDA-approved for locally advanced or metastatic urothelial cancer (2019)
Zynlonta Loncastuximab tesirine-lpyl	CD19	IgG1	Cleavable	SG3199/PBD dimer	DNA cleavage	3.3	Large B-cell lymphoma (April 2021)
Fam- trastuzumab deruxtecan- nxki (T- DXd)	HER2	IgG1	Cleavable	DXd	TOPO1 inhibitor	8	Advanced- stage HER2+ breast cancer after two or more anti- HER2- based regimens (2019)
Tivdak Tisotumab vedotin-tftv	Tissue factor	IgG1	Cleavable	MMAE/auristatin	Microtubule inhibitor	4	Recurrent or metastatic cervical cancer (Sep 2021)
Belantamab mafodotin- blmf	BCMA	IgG1	Non- cleavable	MMAF	Microtubule inhibitor	N/A	R/R multiple myeloma in the fifth-line setting or beyond (2020
Elahere Mirvetuximab soravtansine	FRα	IgG1	Cleavable	Maytansinoid DM4	Folate receptor alpha	N/A	Adult patients with folate receptor-α (FRα)-positive, platinum-resistant epithelial ovarian, fallopian tube or primary peritoneal cancer (Nov 2022)

ADC: The name of the antibody-drug conjugate; Antigen Targeted: The antigen or protein targeted by the antibody component of the ADC; Antibody Isotype: The type of antibody used in the ADC, typically IgG1, IgG4, or a bispecific T cell engager (BiTE); Linker Type: The type of linker used to attach the drug or cytotoxic agent to the antibody; Warhead: The drug or cytotoxic agent component of the ADC; Mechanism of Action: How the ADC targets and kills cancer cells; DAR: The drug-to-antibody ratio, which indicates the average number of warheads per antibody molecule in the ADC; Clinical Usage: The approved or investigational clinical use of the ADC, including the type of cancer or disease targeted.

Several new techniques have been adopted to maximize the therapeutic index by selecting specific groups of patients who may benefit from these next generation ADCs. Besides, development of new more potent warheads free from cytotoxic action have also been actively pursued. It is worth mentioning that LMB-100 (pseudomonas exotoxin A) and ABBV-155 (Bcl-2 inhibitors) are two such proapoptotic payloads under investigational ADCs (81). Further, there are other ADCs that deliver certain immunomodulatory drugs like TLR7/8 alongside with new traditional auristatins and maytansinoids, calicheamicins and camptothecin based analogues. Equal efforts have also been made on improving linker technology to avoid premature release of drug. New approaches are being explored to improve the antibody antigen binding, for example, biparatopic and bispecific mAbs have been used in some preclinical models (82, 83). The biparatopic mAb binds with two non-overlapping antigen epitopes, whereas the bispecific mAb is able to identify two distinct antigen epitopes on the same antigen. Further, to optimize the mAb used for ADC technology, a modified tumor specific mAb, named probody has been used that showed enhanced therapeutic index in an EGFR-overexpressing mouse model. Probody has been modified to mask the Fab paratopes to limit its activity in healthy normal tissue, and use of such probodies in ADCs to enhance tumor cell-specific delivery of drugs has recently been tested with encouraging results (84).

Several current trials are evaluating new ADCs with other payloads, chemotherapeutic agents, or immune checkpoint inhibitors to improve their effectiveness further. It is worth noting that several of these new ADCs act via stimulating immunogenic cell death, potentially increasing therapeutic activity (85). Recently novel entities like Degrader-Antibody Conjugates (DACs) are developed which are the combination of proteolysis targeting chimera (PROTAC) payload conjugated with monoclonal antibodies by a linker molecule. These PROTAC are well established but targeted protein degradation with antibody-PROTAC conjugation is an area of great intrest currently. Trastuzumab-PROTAC conjugate (Ab-PROTAC **3**) that spares HER2 negative cells and only target bromodomain-containing protein 4 (BRD4) in HER2 positive breast cancer cell lines (86, 87).

Despite ongoing advancements in the realm of ADCs, several difficulties still remain elusive with major issues being hazy safety profiles and toxicities mechanisms. These AEs can be induced by on-target/off-target toxicities as well as payload side effects (12). Interestingly it has been observed that most of these ADCs have been shown to work better than their drug counterparts. For instance, despite having same payload and linker structures with comparable DAR, brentuximab vedotin, polatuzumab vedotin and enfortumab vedotin seem to have different toxicity profiles (88). In other cases, HER2- targeted ADCs can cause pulmonary toxicities via an unspecified mechanism and independent of the ADC target, MMAF (monomethyl auristatin F) can cause ocular toxicity, which is absent in MMAE, despite being both use the drugs called auristatins (12). Moreover, uptake of ADC into cells other than tumor cells may also happen via macro or micro-pinocytosis as well as by binding to Fc receptors that further can add to toxicity index (75). Even though certain debilitating or possibly fatal toxicities have been reported, the safety profiles of new ADCs are typically good. As a result, while choosing patients, it is mandatory to consider the toxicity profile of ADCs and all preventive procedures should be performed stringently keeping in mind the possibility of deadly occurrences. The approval and availability of multiple ADCs with diverse structures and payloads suggest that such a dream is achievable and can be the new leap in drug development strategies for cancer and possible other human diseases.

## 8 Conclusions and future

ADCs have shown great potential and have already been established as a strong candidate for targeted cancer therapy with recent success of a few products. Following decades of pre-clinical research and clinical testing, technical advancements and a better mechanistic understanding of ADC action have led to the invention of numerous drugs that give proven therapeutic advantage to cancer patients. To improve the efficacy of an ADC design, each essential component must be evaluated systemically. Furthermore, a greater knowledge of the molecular processes involved in ADC resistance may allow for more rational ADC design and better treatment results. A large number of ADCs are currently in various clinical stages of development, and the results generated by these next generation ADCs will help to get further insights into the mechanistic basis of ADC design as well as the opportunities for improved understanding of the impact of changes in ADC properties on therapeutic activity and safety. Given that fact, ADCs are being considered more and more and many are awaiting FDA approval in the future. Even though ADCs come with their own challenges, new research and further refinement in the construction and delivery system are always being made to address these issues. While the promise of ADCs with more targeted chemotherapy and less toxicity has yet to be achieved, sustained improvements in technology combined with a treasure of clinical data certainly show optimism to shape the future development of ADCs. A large part of research is focused on new system for stabilizing the payload, on developing high level of conjugation, that can make it possible to use less potent cytotoxic drugs than the classical tubulin binder or DNA damaging agents (89–91).

Combination treatments, in particular combinations of medicines whose modes of action overlap with tumor biology, have the potential to increase the therapeutic index of ADCs. Preclinical research, for example, by Immunomedics, established a basis for co-dosing an ADC administered with other small-molecular drugs which can decrease multidrug resistance (MDR) by inhibiting efflux activity, an intriguing mechanism shown by tumor cells (92). Another example at this end is the combination of AXL-107-MMAE and BRAF/MEK, where studies have shown that AXL-107-MMAE alone has minimal impact compared to the AXL-107-MMAE and BRAF/MEK combination (93). Further, applications of ADCs are not just confined to cancers only rather more indications are being added gradually by virtue of its unique properties. For example, in the treatment of illnesses caused by drug-resistant bacteria, an antibody-antibiotic conjugate has been found to be more efficient than the free antibiotic payload. ADCs and similar conjugates may potentially aid in the treatment of chronic illnesses such as autoimmune and cardiovascular diseases by employing selective payload delivery to avoid adverse effects. Based on the advancements already gained and bridging the knowledge gap on critical ADC features such as mAb, payload, linker, and tumor cell biology, a steady improvement in future ADCs will be achieved to usher in a new and exciting age of ADC therapies.

## Author contributions

All authors DA, JY, MAS, SS, MYA, SM, MK, XH and JSP contributed to writing the review. DA, JY and JSP developed the concept, structure, and table. SS developed the structure. MYA and JSP prepared the figures. MAS, XH and JSP did final editing of the review. All authors contributed to the article and approved the submitted version.
